# Vection and visually induced motion sickness: how are they related?

**DOI:** 10.3389/fpsyg.2015.00472

**Published:** 2015-04-20

**Authors:** Behrang Keshavarz, Bernhard E. Riecke, Lawrence J. Hettinger, Jennifer L. Campos

**Affiliations:** ^1^Intelligent Design for Adaptation, Participation and Technology (iDAPT), Research Department, Toronto Rehabilitation Institute, University Health Network, Toronto, ON, Canada; ^2^School of Interactive Arts and Technology, Simon Fraser University, Surrey, BC, Canada; ^3^Center for Behavioral Sciences, Liberty Mutual Research Institute for Safety, Hopkinton, MA, USA; ^4^Department of Psychology, University of Toronto, Toronto, ON, Canada

**Keywords:** vection, illusory self-motion, motion sickness, visually induced motion sickness, sensory conflict

## Abstract

The occurrence of visually induced motion sickness has been frequently linked to the sensation of illusory self-motion (vection), however, the precise nature of this relationship is still not fully understood. To date, it is still a matter of debate as to whether vection is a necessary prerequisite for visually induced motion sickness (VIMS). That is, can there be VIMS without any sensation of self-motion? In this paper, we will describe the possible nature of this relationship, review the literature that addresses this relationship (including theoretical accounts of vection and VIMS), and offer suggestions with respect to operationally defining and reporting these phenomena in future.

## Overview

Vection describes the sensation of illusory self-motion in the absence of physical movement through space (Fischer and Kornmüller, 1930; Dichgans and Brandt, 1973; see also Palmisano et al., 2015, for a discussion of terminology). Vection is a well-known phenomenon and first scientific reports of vection can be traced back to the late nineteenth century (e.g., Mach, 1875; Wood, 1895). A typical real-life example of vection is the train illusion, whereby seeing the movement of a neighboring train creates the illusion that one’s own stationary train is moving. Vection can also readily occur in virtual environments, movie theaters, or simulators (see Hettinger et al., 2014, for an overview).

Another experience that has been associated with illusory self-motion is *visually induced motion sickness (VIMS)*. VIMS is a sensation very similar to traditional motion sickness (MS), with the difference being that physical movement is usually limited or absent during VIMS (see Keshavarz et al., 2014a, for an overview). Typically, VIMS has been used as an umbrella term to describe MS-like symptoms that are strongly driven by visual stimulation in the absence of physical movement. Depending on the equipment and the laboratory setting, VIMS has been further segmented into different subcategories. For instance, VIMS in virtual environments has been labeled as *cybersickness* (e.g., McCauley and Sharkey, 1992), VIMS during video games has been labeled as *gaming sickness* (e.g., Merhi et al., 2007), and VIMS in driving or flight simulators has been labeled as *simulator sickness* (e.g., Brooks et al., 2010). Note, however, that modern simulators can also provide non-visual cues that might induce sickness, such as physical movement. Thus, simulator sickness and cybersickness can include aspects from both VIMS and traditional MS that cannot always be clearly assigned to one of the two.

Despite the different terminology, the symptom cluster for all VIMS subcategories is similar (but not identical). Typical symptoms related to VIMS include drowsiness, dizziness, fatigue, pallor, cold sweat, oculomotor disturbances, nausea, and (rarely) vomiting (e.g., Miller and Graybiel, 1974; Lawson, 2001). Although the symptom cluster of VIMS and MS are generally very similar, oculomotor disturbances and disorientation are more prominent symptoms during VIMS compared to traditional MS (Stanney and Kennedy, 1997; see Lawson, 2014, for an overview). Slight variations between different VIMS subtypes have been mentioned by Stanney et al. (1997). The authors report that simulator sickness varies from cybersickness, with cybersickness resulting in more disorientation (including focusing problems, vertigo, fullness of head, blurred vision) and less nausea (including general discomfort, increased salivation, sweating, and difficulty concentrating).

Several theories try to explain the origin of VIMS. The **sensory conflict theory** (Reason and Brand, 1975; Reason, 1978; Oman, 1990; Bles et al., 1998; Bos et al., 2008) is arguably the most prominent theory used to explain VIMS and proposes that VIMS results from a mismatch between (or within) the visual, vestibular, and somatosensory senses (see Reason and Brand, 1975, p. 108, for different conflict types). For instance, a fixed-based driving simulator might visually indicate self-motion, but the corresponding vestibular and somatosensory inputs that are typically experienced during real-world driving (i.e., during accelerations, braking, or turning) indicate a lack of self-motion. Another prominent theory highlights the potential role of **postural stability** (Riccio and Stoffregen, 1991; Stoffregen and Riccio, 1991), claiming that changes in postural stability are the crucial factor underlying VIMS. Following this approach, postural instability is not only a consequence of VIMS, but also precedes and causes it. In other words, individuals who already demonstrate poor postural control and/or environmental conditions that lead to poor postural control, are thought to introduce a greater risk of VIMS even before the dynamic visual stimulus is presented. Finally, **eye movements** have also been described as the potential root mechanism for VIMS (e.g., Ebenholtz, 1992). According to the eye movement theory, optokinetic nystagmus (OKN) evoked by moving visual patterns can innervate the vagal nerve, which in turn might lead to VIMS. Note that these theories are not necessarily exclusive or exhaustive, that is, elements of each theory may be true at least for certain situations and can interact with each other.

Based on past literature and common experiences, it is clear that vection can be and often is experienced in the complete absence of MS, but what is not clear is whether VIMS can be experienced in the complete absence of vection. That is, is the experience of compelling self-motion a necessary prerequisite for VIMS, or can VIMS be experienced without feeling as though one is truly moving? There are conditions under which purely visual motion stimuli provide information about self-motion (e.g., direction, speed, distance), but do not induce vection. Can these conditions still create VIMS? In other words, the conditions under which dynamic, visual, ego-motion information leads specifically to the negative symptoms associated with VIMS is currently unclear. Complicating matters is the fact that much of the literature aimed at characterizing vection does not explicitly measure MS and *vice versa*. Critically, even those studies that do indeed measure VIMS often do not explicitly report it if VIMS is not a main focus of the study. Further, vection studies typically try to avoid VIMS as this would reduce the reliability and validity of the data. Therefore, it is common for such studies to intentionally exclude participants from further analysis as soon as they experience any adverse symptoms of VIMS (the cut-off criteria for exclusion being highly varied across studies). It is also very difficult to simultaneously measure the time course of vection and VIMS onset, duration, and severity using a combined protocol in a way that will not compromise the conclusions that can be drawn from these individual measures.

The neurocognitive basis of VIMS and vection have been recently studied, however, the precise mechanisms underlying both sensations are not fully understood. Several brain areas are involved during VIMS, including the vestibulocochlear nerve and the vestibular nuclei, sections of the brainstem, the hypothalamus, parts of the cerebellum (uvula and nodulus), the area postrema, the medulla oblongata (nucleus tractus solitaries), and parts of the reticular formation (see Schmäl, 2013; Yates et al., 2014, for overviews). During vection, cortical activity is meant to be increased in various brain areas (see Kovacs et al., 2008; Keshavarz and Berti, 2014; Palmisano et al., 2015), including the visual areas V1–V3, area medial temporal (MT/V5) and medial superior temporal (MST), parietal brain areas (dorsal intraparietal sulcus medial and lateral, posterior intraparietal sulcus, precuneus), frontal brain areas (right central sulcus), and parts of the limbic system (nucleus caudatus). To our knowledge, there is no study that measured neural activity for VIMS and vection simultaneously. Although it is highly desired to understand the processes that may be jointly involved in vection and VIMS, a neurological connection has not yet been established between the two. A potential neurological link between the two phenomena could involve the area of the brain that is meant to be the human “vestibular cortex” (posterior parieto-insular cortex, Guldin and Grüsser, 1998), as this area is not only meant to be involved during VIMS, but also shares connections with the precuneus, which is known to be involved during vection (Kleinschmidt et al., 2002). However, the precise nature of a connection between vection and VIMS remains speculative at this stage and future studies are necessary to investigate whether vection is directly connected to the sensation of VIMS through a shared neural mechanism.

The present paper has three goals: the first goal is to shed some light on the ongoing debate regarding whether vection is mandatory for VIMS. Therefore, we will re-visit the relationship between vection and VIMS by discussing the role of vection within the concept of different VIMS theories and by summarizing the most relevant (albeit limited) empirical findings in the existing literature. The second goal of this paper is to discuss the implications of vection and VIMS on simulation applications and research programs that involve illusory self-motion. This becomes increasingly relevant given that simulators are becoming very powerful tools for use in occupational/military training, rehabilitation, and entertainment applications and have become accessible to a broad range of populations. As most simulators aim to be highly immersive and to provide an experience that closely mimics reality, vection is often a desirable sensation. However, this may necessarily be at the expense of also experiencing some degree of VIMS. For example, recent developments in low-cost head-mounted displays that offer a wide field of view have led to increasing reports of not only vection but also VIMS, which has raised concerns regarding their usability and safety. Thus, it is not only theoretically interesting but also increasingly practically relevant to better understand how to reduce the occurrence and magnitude of VIMS in contexts which may necessarily simulate self-motion to varying degrees. Finally, the third goal is to make recommendations regarding the future of vection and VIMS research.

## Highlights from Empirical Evidence Directly Comparing Vection and VIMS

Finding reliable measures of VIMS in vection studies is complicated by the fact that most vection studies do not report VIMS data, even if they might be monitoring VIMS during the experiment to ensure that participants did not get sick and to have confidence that the vection data is valid (i.e., not compromised by adverse side effects). It is common practice to typically exclude participants who report VIMS from vection studies and abstain from reporting the VIMS data. In this section we only describe studies that simultaneously measured vection and VIMS and reported both of these datasets across participants. The numerous studies that record vection but not VIMS (or that do not explicitly report the VIMS data) are not included here.

In one of the first studies to jointly address VIMS and vection, Hettinger et al. (1990) exposed their participants to a fixed-based flight simulator and collected both subjective vection and VIMS ratings. Their results showed that eight of nine participants who reported VIMS also reported vection. On the other hand, of the five participants who did not experience vection, only one reported VIMS. These findings indicate that most of the participants who reported VIMS also reported vection. Although the authors state that “…visual displays that produce vection are more likely to produce simulator sickness” (p. 179), and their results showed that VIMS and vection typically (but not always) occur simultaneously, vection has been presumed to be a prerequisite for VIMS. In support of the notion that VIMS and vection are related, Smart et al. (2002) moved a furnished room back and forth around stationary participants and found that 11 of 12 participants reported vection and three of the 11 participants who experienced vection also reported VIMS. The authors used this as evidence to suggest that vection is not only involved in the occurrence of VIMS, but rather a prerequisite for it. Given that only one participant did not experience vection, this argument seems not particularly compelling. In a recent study, Keshavarz et al. (2014b) compared the contributions of dynamic auditory and visual inputs (individually and combined) to vection ratings and VIMS. In this study, participants were seated in a stationary position surrounded by a large, immersive, curved projection display and were exposed to a rotating stimulus that contained either only visual, only auditory, or a combination of both auditory and visual information. Additionally, participants performed head movements (alternately tilting the head to the right or left shoulder) while watching the rotating stimulus to create pseudo-Coriolis sensations and to encourage the likelihood of VIMS. Most participants in the visual-only and the combined audio-visual condition reported VIMS and all of these participants also experienced vection. Interestingly, pure auditory stimulation created sickness in two participants and again, both participants also reported vection. These and other results (e.g., Lee et al., 1997; Stoffregen and Smart, 1998; Classen et al., 2011) indicate that the occurrence of VIMS is tightly linked to the occurrence of vection, in the sense that VIMS does not seem to occur in participants who do not perceive vection. This is consistent with the notion that vection might be a ***necessary prerequisite*** for VIMS to occur. However, as the same studies also show that many participants who reported vection do not experience VIMS, vection alone is clearly ***not a sufficient*** condition for VIMS to occur (e.g., Hettinger et al., 1990; Smart et al., 2002; Keshavarz et al., 2014b). Instead, other factors—such as intra-individual differences (e.g., age, sex), the amount/magnitude of the sensory conflict, the type of motion profile, or the history of perceptual-motor congruency within that particular context, likely relate to the probability of experiencing VIMS (see Diels and Howarth, 2011).

If vection is causally related to VIMS and a determinant of the occurrence and/or strength of VIMS, one might predict that the strength of VIMS would be positively correlated with the strength of vection. That is, conditions that induce stronger vection should also induce stronger VIMS and *vice versa*. Several studies have now demonstrated positive correlations between vection and VIMS to support this assertion (e.g., Bonato et al., 2004, 2005, 2008; Flanagan et al., 2004; Bubka et al., 2006; Golding et al., 2009; Diels and Howarth, 2011), whereas others have failed to find support (Lawson, 2005; Golding et al., 2012; Keshavarz et al., 2014c). For instance, Diels et al. (2007) displayed a contracting or expanding optic flow field (random dots) on a wide field-of-view screen and measured subjective ratings of VIMS and vection. The reported high positive correlations (up to *r* = 0.70) indicated that participants who reported stronger VIMS also reported stronger vection. Moderate and high positive correlations were also reported by Palmisano et al. (2007), who added viewpoint jitter to a constantly expanding flow field, which both increased the level of vection and increased ratings of VIMS. In a recent study, Keshavarz and Berti (2014) collected subjective ratings of vection and VIMS from participants who were exposed to horizontally moving patterns of altered black-and-white stripes. Again, a moderate positive correlation (*r* = 0.47) was shown, although it was not statistically significant.

On the other hand, several studies have not observed a positive correlation between vection and VIMS and in fact, some have even observed negative correlations. For instance, Keshavarz et al. (2014c) exposed participants to a rotating stimulus that caused both vection and VIMS. Although all participants who reported VIMS also reported vection, only weak correlations (up to *r* = 0.20) between subjective ratings of vection and VIMS were observed (see also Lawson, 2005; Keshavarz and Hecht, 2011b; Golding et al., 2012).

Further evidence indicating that the magnitude of VIMS is not determined by the magnitude of vection is given by studies comparing conditions of different vection-inducing or VIMS-inducing stimuli. For example, Bonato et al. (2009; see also Chen et al., 2011) exposed their participants to a video containing optic flow rotations along a single axis or along a combination of two axes. Although dual-axes rotations elicited more VIMS, the level of vection remained unchanged compared to the single-axis condition. Similar results were reported by Keshavarz and Hecht (2011a), who also found more VIMS when participants watched a video that contained rotations along two or three axes compared to a single axis, but failed to find differences in vection ratings. In another study, Prothero et al. (1999) introduced an independent visual background to reduce VIMS. The independent visual background consisted of an array of fixed horizontal and vertical lines that were visually superimposed on top of a visual image, similar to a grid. This independent background was stable and did not move, thus it was used as a reference for the participants’ stationary position. The authors found that the introduction of the independent background significantly reduced the level of VIMS compared to no independent background, whereas the level of vection was unchanged. Finally, Webb and Griffin (2003) tested the level of VIMS and vection experienced when participants were exposed to optic flow displays of varying densities and found that a higher number of moving dots (i.e., stronger optic flow) increased the level of vection, but did not change VIMS severity.

Up until this point it is clear that VIMS is typically accompanied by vection, but that vection can clearly be experienced in the absence of VIMS. However, to answer the question of whether vection is a necessary prerequisite for VIMS in the sense that VIMS cannot occur without vection, it is important to create a scenario where VIMS can be experienced without vection. To our knowledge, Ji et al. (2009) are the only ones to describe data to empirically support this contention. Specifically, the authors displayed a pattern of alternating black-and-white horizontal stripes that rotated around a stationary observer seated in front of a curved projection screen. The stimulus pattern was divided into a central, ellipse-shaped field and a peripheral field. Both fields moved independently from each other, that is, the center and periphery moved either in the same or in the opposite direction. The authors found that vection was not experienced when the peripheral and the central stimulus moved in opposite directions. However, this did not affect the level of VIMS reported by the participants (measured via a 7-point nausea rating scale); instead, VIMS severity was similar when the pattern moved in the same or opposite direction. The authors postulate that VIMS can therefore be experienced without vection and thus, vection is not a necessary prerequisite for VIMS. The study by Ji et al. (2009) is doubtlessly the first step toward solving the controversy regarding the relationship between vection and VIMS.

## Reflection on the Relationship between Vection and VIMS from Different VIMS-Specific Theoretical Approaches

### Sensory Conflict Theory

The sensory conflict theory postulates that the dominant causes of VIMS are mismatches between (or within) the visual, vestibular, and somatosensory inputs (Reason and Brand, 1975; Reason, 1978; Oman, 1990; Bles et al., 1998; Bos et al., 2008). Because physical movement is typically missing or limited during VIMS, it is often assumed that the visual information that is specifying self-motion conflicts with the lack of self-motion specified by the physical cues, resulting in a sensory mismatch that causes adverse symptoms. However, if the visual stimulus fails to create the sensation of vection (even though a sensory conflict is still present), does VIMS still occur? In all known accounts of the sensory conflict theory, there is no conclusive claim that vection is necessary to experience VIMS.

While increasing the visuo-vestibular cue conflict tends to enhance VIMS, it does not necessarily decrease the level of vection. In fact, vection has been shown to be facilitated when the sensory conflict is *reduced*, whereas VIMS tends to be stronger for *increased* sensory conflict, which seems to contradict the notion of a causal relationship. Notably, vection often does not occur instantaneously with the onset of full-field visual motion, but only after a certain onset latency. This is thought to be due to an initial inter-sensory cue conflict during the acceleration phase between the visual stimuli specifying self-motion (e.g., optic flow) and the non-visual stimuli specifying a lack of self-motion (i.e., vestibular cues). In support of this notion, bilaterally labyrinthine defective participants (where visuo-vestibular conflicts are largely non-existent) were found to perceive visually-induced vection much earlier and more intensely (Johnson et al., 1999).

Since the vestibular apparatus is only sensitive to *changes* in velocity (acceleration/deceleration), the brain does not expect vestibular feedback during constant velocity motion. Therefore, if the dynamic visual display contains only constant velocity motion or sub-threshold accelerations, there should be little or no sustained conflict between the visual and vestibular inputs apart from the initial acceleration phase. If the visuals are compelling, this would introduce the impression of illusory self-motion without introducing a sensory conflict, and would likely not induce any VIMS. However, if additional accelerations are introduced, such as tilting the head along the roll or pitch axes while experiencing constant forward vection, this mismatch between the vestibular and the visual system introduces a sensory conflict, and VIMS can occur as a result. In fact, such head-movements have been demonstrated to cause so-called pseudo-Coriolis effects and are typically a powerful stimulus to cause VIMS (see Dichgans and Brandt, 1973; Keshavarz et al., 2014b). Similarly, Bonato et al. (2008) compared two groups of participants: the first group was exposed to a constantly expanding optic flow field (less visual-vestibular conflict due to a lack of acceleration in the motion profile), whereas the second group was exposed to an optic flow field that alternately expanded or contracted (more visual-vestibular conflict due to frequent changes in velocity). Vection onset, vection duration, vection strength and VIMS were verbally reported. Results showed that the second group reported significantly less vection in total, more frequent changes in vection (i.e., alternating vection onset and offset), and increased VIMS, whereas the first group experienced more compelling and longer-lasting feelings of vection and significantly reduced VIMS. Again, this finding is in accordance with the idea that sensory conflict might relate more directly to causing VIMS, but does not necessarily enhance vection (in fact, it may result in reductions in vection). Support for this assumption is also provided by Palmisano et al. (2009), who added visual jitter to a stimulus that created forward vection. Despite increasing the sensory conflict by adding the visual jitter, results showed stronger vection than the same visual stimulus without jitter. However, no sickness data were reported in this study.

## Drawing from Theories and Approaches in the Context of Multisensory Integration

As described above, one of the most typically assumed causes of MS in general and VIMS in particular is the sensory conflict that occurs between visual and non-visual feedback. Vection, by definition, is illusory self-motion in the absence of physical self-motion through space and thus, is inherently an experience that occurs under sensory conflict conditions. However, the nature of the conflict and the magnitude of the conflict can range along a continuum (i.e., strong inter-sensory conflict vs. subtle inter-sensory conflict), which presumably also leads to different subjective experiences and behavioral effects. Accordingly, depending on the precise nature of the conflict, this may result in varying levels of vection and varying levels of VIMS, and may lead to one in the presence or absence of the other.

We can consider these issues in light of prominent concepts and models in the multisensory literature, which often intentionally adopt “cue conflict” approaches to quantify the relative weighting and integration of individual sensory signals or to better understand the effects of combining sensory inputs. For instance, sensory “capture” is thought to occur when, under multisensory conditions (e.g., both seeing and hearing an event), one sensory stimulus (e.g., vision) completely dominates the percept (e.g., Rock and Victor, 1964). For example, in the ventriloquist effect, visual stimuli are thought to “capture” the perceived location of the associated auditory stimulus in order to combine temporally and semantically coincident multisensory input into a unified percept. Under conditions in which one sensory input is simply ignored in favor of the dominant sensory cue, this is often referred to as a “winner takes all” strategy (e.g., Mulligan and Shaw, 1980). Further, a prominent and well-supported model of multisensory integration is the Maximum Likelihood Estimation model (Ernst and Banks, 2002; Alais and Burr, 2004; Ernst and Bülthoff, 2004). This model specifies that the brain uses a weighted sum of two or more sensory inputs with the individual sensory weights reflecting individual sensory reliabilities; the more reliable (less variable) input being weighted higher in the final estimate. Notably, optimal integration should only occur if the sensory events occur together within close temporal or spatial proximity (i.e., within an acceptable spatio-temporal “binding window”—see Wallace et al., 2004). Evidence in support of this model has been provided at both behavioral and neurophysiological levels (Stein and Meredith, 1993; Stein et al., 2014), and across a variety of sensory combinations, with some of the most recent evidence being in the context of visual-vestibular integration (Gu et al., 2008; Fetsch et al., 2009; Butler et al., 2010, 2014).

Moving forward, we should now apply these models to help reconcile conditions under which vection is experienced in the presence of VIMS vs. in the absence of VIMS. For instance, in the context of self-motion, under conditions in which one sensory input is particularly reliable and other modalities are much less reliable, the brain may ignore the most unreliable conflicting cue (winner takes all), or the more reliable cue may capture the percept. Under such conditions, one might experience a compelling sense of vection, but not experience VIMS, because the sensory conflict is easy to reconcile or ignore. In contrast, when there are cue conflicts and the cues are equally reliable, it is possible that the brain continues to attempt to integrate these signals despite the fact that they are in conflict. If the conflict is small enough, optimal weighting may lead to vection in the absence of VIMS. However, if the conflict is of a certain magnitude that exceeds some threshold, it is possible that the brain is unable to either completely ignore or optimally integrate these signals. It may be under these circumstances that one can experience both vection and VIMS (or potentially VIMS in the absence of vection).

Importantly, despite the fact that these predictions are based on established theoretical constructs, they have not been tested empirically in the context of vection and VIMS and in defining their relationship. Therefore, future research may benefit from adopting a formalized approach of experimentally testing and modeling the data based on these predictions.

### Postural Instability Theory

The postural in stability theory of VIMS suggests that the occurrence of VIMS is mainly caused by changes in postural stability (Riccio and Stoffregen, 1991; Stoffregen and Riccio, 1991). Specifically, the ability to maintain postural control is decreased in challenging situations that involve (real or virtual) self-motion cues, such as is the case when traveling by car, train, or ship or when being exposed to compelling, global, dynamic visual cues. The changes in postural stability are meant to precede the onset of VIMS and to be the cause for and not only a by-product of VIMS (e.g., Stoffregen et al., 2000; Smart et al., 2002; Flanagan et al., 2004; Reed-Jones et al., 2008; Villard et al., 2008). Although this theory of VIMS does not explicitly describe a role for vection, previous studies have shown that vection and postural sway may be linked (see Lestienne et al., 1977; Mergner et al., 2005; Wei et al., 2010). Kuno et al. (1999) found that postural responses were observed immediately *after* participants reported vection, not only indicating a positive correlation between vection and postural sway, but also highlighting the temporal relation between the two concepts, suggesting that vection might precede or trigger postural sway (see also Berthoz et al., 1975). Other studies have proposed that postural sway and vection share common underlying neural mechanisms (Previc and Mullen, 1990; Tanahashi et al., 2007). Recently, Palmisano et al. (2014) found that postural instability can precede vection, that is, postural stability can be used to predict the sensitivity or predisposition to vection. The authors tested participants’ postural stability prior to being exposed to a vection-inducing stimulus. Results showed that participants who had more spontaneous sway before stimulus exposure reported stronger vection during stimulus exposure. However, no information on VIMS was reported as a part of this study. In another study, Fushiki et al. (2005) exposed their participants to an upward or downward moving random dot pattern and measured vection onset times and postural stability before, during, and after stimulus exposure. Results showed that postural sway was increased once participants reported vection. Also, postural sway was stronger after stimulus presentation compared to prior to stimulus onset. This seems to suggest that if vection is related to increased postural instability and postural instability is related to increased MS, an indirect relationship between vection and VIMS under some conditions could be possible. However, this is clearly not a causal relationship given that perception of vection would need to *always* precede the occurrence of postural instability, which is not the case. In fact, postural instability can occur well before the onset of vection and is not necessarily coincident or consistent with the direction of perceived self-motion (Guerraz and Bronstein, 2008; Wang et al., 2010). Guerraz and Bronstein (2008) suggest that there might, however, be additional longer-latency postural response mechanisms that are influenced by the (conscious) perception of self-motion.

### Eye Movement Hypothesis

The eye-movement theory by Ebenholtz (1992) proposes that OKN evoked by moving visual patterns innervates the vagal nerve and such innervations leads to VIMS. For instance, Hu et al. (1989) exposed their participants to different visual rotational velocities within an optokinetic drum, thereby varying the amount of OKN and reported that VIMS severity increased when the drum rotated faster (note that faster drum rotations also caused a stronger sensory conflict and potentially changes in postural stability as well). Other studies introduced a fixation point to reduce eye movements during stimulus presentation and succeeded in decreasing VIMS (Flanagan et al., 2002, 2004; Webb and Griffin, 2002). The role of vection within the eye-movement theory is controversial. On the one hand, vection might be an important factor mediating the relationship between OKN and VIMS in most studies. Decreasing the velocity of a rotating drum, for example, does not only reduce OKN, but also tends to decrease the level of vection (e.g., Bubka et al., 2006). On the other hand, introducing a fixation point reduces both OKN and VIMS but not vection strength (Riecke et al., 2004; Ji et al., 2009). Instead, a fixation point tends to actually increase vection (Fischer and Kornmüller, 1930; Becker et al., 2002), contradicting the assumption that vection might be the main component causing VIMS. The eye-movement theory might also explain why time delays and spatial asynchronies that are often a characteristic of head-mounted displays can cause dizziness and vertigo without eliciting vection.

## Challenges in Addressing the Relationship between Vection and VIMS

### Measuring Vection and VIMS

Subjective ratings are the most common method of collecting both vection and VIMS data (i.e., onset time, strength, duration). However, subjective ratings have various shortcomings, such as a lack of reliability, response biases, or social desirability effects (see Podsakoff et al., 2003). Vection strength, for instance, is commonly measured using rating scales (e.g., see Riecke et al., 2009a,b, 2015; Keshavarz et al., 2014b,c). The definition of vection is of particular importance when using such scales, that is, participants need to know what is *meant* by “fully saturated vection,” and more importantly, what fully saturated vection *feels* like. It is extremely difficult to verbally explain this experiential phenomenon and to operationalize a “strength” metric that is intuitive and consistent for most people. In order to address this concern, some studies have used practice trials that are intended to induce strong and saturated vection prior to the experimental session as a way of familiarizing participants with the concept of vection and what saturated vection feels like (e.g., Riecke et al., 2009b, 2015; Riecke and Feuereissen, 2012). Surprisingly, the use of such practice trials is anything but a standard procedure in most vection research. Thus, most subjective vection ratings have to be treated carefully and the comparison of vection data across studies is very difficult. Meta-analyses of such studies, which, to our knowledge, have not been conducted, could be seriously limited by this issue. Similarly, subjective ratings are also the method of choice to measure VIMS. Compared to vection research, standardized subjective rating questionnaires for VIMS do exist, including the prominent Simulator Sickness Questionnaire (Kennedy et al., 1993), the Fast Motion Sickness Scale (Keshavarz and Hecht, 2011b), or the Misery Scale (Bos et al., 2010). Note, however, that these measures, although standardized, suffer from similar shortcomings, and it can be difficult to compare absolute VIMS ratings between participants, sessions, or studies.

Several researchers have tried to establish more objective measures of VIMS and vection, such as physiological parameters (e.g., heart rate, electrodermal activity, gastrointestinal activity, etc.). However, none of these parameters can reliably and sufficiently measure the subjective sensation of VIMS (see Shupak and Gordon, 2006, for a discussion) or vection, which are inherently subjective phenomena and experiences. Other studies have aimed to localize neural mechanisms that are involved during vection (e.g., Thilo et al., 2003; Kovacs et al., 2008). These studies found neural areas that are specific to the occurrence of vection, such as the motion-sensitive parts of the occipital lobe (area V5/MT), the precuneus, and the posterior parieto-insular cortex, an area thought to represent the “vestibular cortex” of the human brain. Recently, Keshavarz and Berti (2014) exposed their participants to different patterns of horizontally moving stripes that elicited vection. Results showed that the pattern that created strongest vection also showed pronounced event-related brain potentials in the occipital lobe around 230 ms after stimulus onset (N2), indicating that the N2 mirrored the subjective rating of vection strength. As the N2 occurs long before the subjective onset of vection, the authors argue that event-related brain potentials can presumably precede the onset of vection. Overall, given that it is still very difficult to objectively measure vection and VIMS individually, empirically comparing these experiences in a way that helps to better define their relationship is an even greater challenge.

### Characterization of Vection and VIMS Symptoms

Is MS in the absence of real or illusory motion possible? Strictly speaking, we believe that the term *VIMS* indicates the involvement of at least some kind of motion. It is doubtlessly true that other non-motion stimuli, events, or environmental conditions can induce MS-like symptoms such as nausea or discomfort (e.g., unpleasant odors, medication, flickering lights). However, these phenomena are less likely to be categorized as MS *per se*. For instance, Chen et al. (2014) analyzed postural sway and nausea in female boxers after a boxing match and reported that some boxers (most of them who lost their match) reported symptoms matching those of MS. While it is perhaps not surprising that boxers might suffer from dizziness and nausea after a match, it is unlikely that these symptoms would be intuitively attributable to MS *per se*, rather than being an artifact of the physical insult itself. This is one example of how the term MS is sometimes used too liberally when specific symptoms would be better reported as such rather than being classified as MS *per se*. It is important from both theoretical and applied perspectives to be more precise when describing specific symptoms and perceptual sub-categories. For instance, standard measures of VIMS such as the simulator sickness questionnaire (SSQ) ask participants to rate their experiences on several different sub-scales including disorientation, oculomotor disturbances, and nausea. However, presumably each of these groups of symptoms could be rooted in different mechanisms. Some of these sub-categories could be strongly related to vection-inducing stimuli and conditions, whereas others might be related to other characteristics of the stimuli and conditions (e.g., slow display refresh rates). Importantly, despite the existence of these different sub-scores, the SSQ is often reported as one compiled score, thereby potentially masking the role of each symptom category.

As previously mentioned, a study by Ji et al. (2009) showed that OKN elicited discomfort and dizziness in the absence of vection, indicating that vection is not a necessary prerequisite for VIMS-like symptoms. Although this result shows that VIMS-like symptoms can be experienced without vection, it remains to be answered whether these symptoms do in fact represent the syndrome *MS* or are rather symptoms specific to oculomotor disturbances in particular. For example, it has been observed that helmet-mounted displays can introduce spatiotemporal lags and asynchronies in response to head movements, which can lead to significant “motion sickness-like symptoms,” without an associated experience of vection (e.g., DiZio and Lackner, 1997). Other characteristics of visual stimulations such as flickering lights can also elicit dizziness and oculomotor issues (Ulett, 1953; Rash, 2004), in the absence of perceived self-motion. However, the question is whether the label VIMS is appropriate for these cases?

In defining the relationship between VIMS and vection, it will be more constructive to start distinguishing whether visually induced sickness symptoms stem from visually induced *self-motion* or whether it is likely related to other aspects of the stimulus. For instance, flickering stimuli can clearly be categorized as capable of triggering visual discomfort, but they would not be categorized as triggering visually induced motion sickness (VIMS). Overall, there is a clear need to be more precise when measuring and reporting specific symptoms of VIMS and to establish more descriptive categorizations of the subjective experiences associated with the presentation of stimuli intended to induce vection.

## Implications for Application

The quality of simulator systems and virtual reality interfaces has been steadily increasing over the past decades in terms of the graphics, the level of immersion, the capacity to present multi-modal/multisensory experiences, and the ability to physically interact with the simulation in a number of ways. These simulators are used in diverse fields such as research, occupational and military training (see Rizzo et al., 2014), rehabilitation, and by the entertainment industry (see Greenwood-Ericksen et al., 2014) and are often designed to provide a highly compelling and realistic simulation experience. Hence, vection is often a desired phenomenon and can, in fact, be important for these applications (see Palmisano et al., 2015, for an overview). For instance, vection is highly associated with the experience of presence (see Chertoff and Schatz, 2014), and presence is a desired sensation in most virtual reality (VR) settings. Vection can also help to improve task performance as indicated by Riecke et al. (2012) in a perspective taking task. In the case of simulations involving self-motion, the challenge becomes maximizing the experience of vection, while minimizing the occurrence of VIMS. Indeed, if any of the above-mentioned applications were to suffer from high levels of VIMS, simulation technologies within this context would become unusable. In the context of research studies, VIMS can lead to high rates of participant attrition and may compromise the conclusions than can be drawn from experimental outcome measures. From an ethical point of view, exposing participants to unpleasant conditions should obviously also be avoided, even if VIMS is a temporary state and typically fades out shortly after stimulus offset (but see Stanney et al., 1999; Keshavarz and Hecht, 2012, for after-effects of VIMS). Thus, it is clear that reducing or ideally completely avoiding any negative side-effects like VIMS is critical for most situations in both research and applications. Improving our understanding of the relationship between vection and VIMS could be beneficial here, in that it could help to inform the best methods of creating a compelling sensation of self-motion and immersion while avoiding negative side-effects like VIMS.

Given that displays capable of inducing vection can also be prone to induce vection, a crucial point is whether or not vection is necessary or desired for a given application or research question. Self-motion information can be processed within simulated environments without necessarily causing vection. For example, playing a 3D computer game on a small display like a smart phone or desktop monitor can provide users with a photorealistic simulation of moving through a 3D environment but will rarely evoke any embodied percept of actual self-motion. As a consequence, however, realism, immersion, and presence are also likely reduced. So the question is, for which situations is the subjective experience of vection necessary? For instance, vection might be critical to more closely reflect real world behavior, or to allow for optimal “transfer of training” in pilot or driver training. In situations where vection is critical, it becomes important to maintain vection whenever possible while still reducing the co-occurrence of VIMS as much as possible. In contrast, for situations where vection is unnecessary, it might be better to avoid vection if it is likely to also have a higher risk of causing VIMS. For example, large-field-of-view visual displays can induce strong vection, but also tend to potentially induce VIMS. Thus, avoiding such displays can be an effective way of preventing VIMS. Importantly, however, it is currently unknown as to which applications vection is critical and for which it is not.

An approach that has shown promise for maintaining strong vection in virtual environments and to simultaneously keep VIMS to a minimum is to implement *training* or *habituation protocols*. For instance, Kennedy et al. (2000) and others (e.g., Hu et al., 1991) have shown that VIMS is significantly reduced with repeated exposure. In contrast, vection strength is meant to remain robust and not to decrease due to habituation or adaptation. Anecdotal reports, in fact, suggest that vection onset is even reduced and that vection saturates faster when the same vection-inducing stimulus is presented repeatedly. With respect to VIMS, Domeyer et al. (2013) used a step-wise protocol to familiarize participants with a novel driving simulator experience. The authors used an initial 10 min-long acclimation session that familiarized participants with the driving simulator prior to the driving test. The acclimation session was separated into four parts: in the first part, speed and steering were software-driven and pre-defined by the simulator (i.e., participants were not actively involved in the driving procedure). In the second part, participants steered the vehicle while speed was controlled by the simulator. In the third part, participants were in full control of the vehicle but drove only straight sections without turns. In the fourth part, participants were in full control of the vehicle and performed turning. The acclimation session was used for all participants, however, only half of the participants performed the actual driving test immediately after the training session, whereas the other half performed the driving test with a delay of 1 day and after finishing a second acclimation session. Results showed that the group with a delay of at least 24 h between the acclimation session and the driving test reported significantly less VIMS compared to the group who immediately completed the driving test following the first acclimation session. The results by Domeyer et al. (2013) show that a well-designed training protocol can be effective in reducing VIMS. However, note that an extended training protocol as proposed by Domeyer et al. (2013) can be highly time-consuming and cost-intensive and therefore is rarely practical in many laboratories.

Overall, a main challenge will be to truly understand how, whether, and in which contexts the experience of vection is of any consequence to the main outcomes of interest. If vection is indeed desirable or required, a deeper understanding of factors affecting vection and VIMS and their potential interrelations can help to more effectively optimize vection while reducing undesirable side-effects like VIMS.

## Future Directions and Recommended Approaches

The relationship between vection and VIMS has been widely discussed for more than two decades and nonetheless, many questions are still left unanswered and require further empirical consideration. In light of the current review we make several recommendations for future research in this area.

### More Studies that Address Vection and VIMS Simultaneously

Studies directly addressing the relationship between vection and MS are surprisingly sparse. Typically, most studies focus on vection or VIMS, but only rarely are ratings for both collected, reported, and analyzed. This could either be due to non-significant findings, or if these were used as a criteria for eliminating individual participant data. Of those that do collect and report both VIMS and vection data, the different studies often use different measurement procedures, metrics, and experimental protocols (e.g., with or without practice trials, anchoring the scales that measure vection or VIMS, etc.). Thus, one of the biggest challenges is thereby the lack of ability to compare results across studies. Future studies that directly experimentally manipulate and/or measure vection and VIMS using the same protocol within the same participants are highly desirable. We would also urge investigators to be very transparent in reporting these measures routinely. To answer the question of whether vection is a necessary prerequisite for VIMS or not, empirical strategies must be used to decouple vection from VIMS by revealing conditions under which VIMS is induced in the absence of vection. While a first attempt has been made by Ji et al. (2009) as described above, further studies are needed to support and generalize their results.

### The Nomenclature of Vection and VIMS is too Vague

Although some definitions for vection and VIMS have been established in the past, inconsistencies regarding the use of the terms *vection* and *VIMS* still exist. For instance, VIMS is used as a generic term to describe MS-like symptoms where visual stimulation is the main source of sensory feedback. Depending on the application, VIMS has also been labeled as Cinerama sickness (VIMS in movie theaters), cybersickness (VIMS in VR users), simulator sickness (VIMS in driving or flight simulators), or gaming sickness (VIMS during video game playing). Although Stanney and Kennedy (1997) highlighted some differences between simulator sickness and cybersickness, the different VIMS sub-categories seem to be used widely interchangeably and their symptoms are typically described as similar. It is therefore important to operationally define, measure, and report the different types of VIMS and to better describe and characterized subjective experiences associated with vection-inducing stimuli.

As a first step to address this issue, distinguishing between sickness symptoms originating from visually induced (*self*)motion versus other aspects of the stimulus might be helpful. For example, flickering stimuli can be categorized as capable of eliciting VIMS-like symptoms, such as visual eye-strain or visual fatigue, but as they do not simulate any self-motion by any means, it remains questionable whether VIMS is the proper label for these cases.

## Summary

1. Vection can be experienced without VIMS, indicating that vection alone is not a **sufficient prerequisite** for VIMS.
2. The reported experience of VIMS is in many situations associated with a greater likelihood of also experiencing vection, indicating that vection might be a **necessary prerequisite** for VIMS, but only in combination with other factors (e.g., sensory conflict, postural stability, eye-movements, head movements etc.).
3. VIMS-like symptoms (e.g., visual fatigue, eye strain etc.) can occur without corresponding experiences of vection, but *VIMS* might not be the most appropriate term for such events.
4. The relationship between VIMS and vection may be dictated by the magnitude and type of sensory conflict that is present.
5. Novel, more consistent, and reliable methods of quantifying vection and VIMS and a greater transparency with which they are reported are necessary.

### Conflict of Interest Statement

The authors declare that the research was conducted in the absence of any commercial or financial relationships that could be construed as a potential conflict of interest.
